# Unicentric Castleman Disease of the Mesentery: A Systematic Review

**DOI:** 10.7759/cureus.105897

**Published:** 2026-03-26

**Authors:** Jagani Harsha Sai, Ravi Gupta, Vishakha Gupta, Santosh Reddy Baddala, Twvisham Srivastava, Divya Mishra, Aditya Gaurav, Aman Siloiya

**Affiliations:** 1 General Surgery, All India Institute of Medical Sciences, Gorakhpur, IND; 2 Pathology, Sanjay Gandhi Postgraduate Institute of Medical Sciences, Lucknow, IND

**Keywords:** a systematic review, lymphoproliferative malignancy, mesenteric castleman disease, mesenteric tumor, unicentric castleman disease

## Abstract

Castleman disease (CD) is a rare lymphoproliferative disorder categorized into unicentric and multicentric forms. The unicentric form usually manifests as a localized lymph node enlargement. Mesenteric involvement is rare and can mimic other intra-abdominal neoplasms such as gastrointestinal stromal tumors, lymphoma, or metastatic disease, making preoperative diagnosis difficult. The study aimed to systematically analyze published cases of mesenteric unicentric CD (UCD) and evaluate demographic trends, anatomical distribution, histopathological variants, tumor characteristics, treatment strategies, and clinical outcomes. A systematic review of individual case reports published between 1996 and 2026 was performed following the Preferred Reporting Items for Systematic Reviews and Meta-Analyses (PRISMA) framework. The literature search was conducted using PubMed and Scopus as the primary databases. Reports describing mesenteric UCD were screened, and relevant details, including age, sex, lesion size, location, histopathology, and management, were extracted. Data were summarized using descriptive statistics and synthesized narratively. The mean age at diagnosis was 35.3 ± 17.9 years, and the mean lesion size was 5.51 ± 2.25 cm. The mesentery (unspecified) was the most frequently reported site (75.68%), followed by the small bowel mesentery (18.92%) and large bowel mesentery (5.41%). The prevalence of mesenteric UCD with a hyaline vascular variant was 64.86%, followed by plasma cell (21.62%) and mixed types (12.16%). In 1.35% of cases, histology was not specified. Complete surgical excision represented the primary therapeutic approach (60.81%) and was associated with favorable outcomes in most patients. For mesenteric UCD, surgical excision remains the cornerstone of treatment, offering an excellent prognosis. Early recognition and accurate histopathological diagnosis are essential to avoid misclassification and guide optimal management.

## Introduction and background

Castleman disease (CD) consists of a heterogeneous group of lymphoproliferative disorders, classified into unicentric CD (UCD) and multicentric CD (MCD) variants. UCD commonly involves a single lymph node or nodal region and is often asymptomatic or presents with non-specific symptoms [1]. In contrast, MCD presents with constitutional symptoms, and it is a systemic disease involving multiple organs. The mediastinum is the most common site of UCD, accounting for approximately 70% of cases, followed by the intra-abdominal (12%) and cervical regions [1]. Intra-abdominal presentations include retroperitoneum and mesenteric location, of which isolated mesenteric location is rare [2,3]. The disease represents a benign but atypical lymphoid proliferation that can simulate malignant tumors on imaging and clinical evaluation [4]. Radiologically, mesenteric UCD typically appears as a well-circumscribed, hypervascular mass on contrast-enhanced computed tomography with intense enhancement in the arterial phase, mimicking malignant neoplasms such as gastrointestinal stromal tumor, lymphoma, neuroendocrine tumor, or metastatic disease, so preoperative diagnosis is often challenging, and definitive diagnosis is usually established only after surgical excision and histopathological examination [5]. In the literature, the majority of research has been published regarding CD as case reports and case series to know the pattern behind the anatomic distribution, pathological type, management, and prognosis, but a thorough systematic review is needed. Thus, we conducted a systematic review on UCD with mesenteric location.

## Review

Material and methods

Data Source and Searches

We conducted our study in accordance with the Preferred Reporting Items for Systematic Reviews and Meta-Analyses (PRISMA) protocol. A comprehensive literature search was performed using two electronic databases: PubMed and Scopus. Publications describing the UCD of the mesenteric location were targeted. For PubMed, the search strategy was ("Castleman Disease"[MeSH] OR "unicentric Castleman disease"[All Fields] OR "localized Castleman"[All Fields]) AND ("Mesentery"[MeSH] OR mesenteric[All Fields] OR "mesenteric mass"[All Fields] OR "mesenteric lymph node"[All Fields]) AND (English[Filter]) AND ("1996/01/01"[Date - Publication] : "2026/02/27"[Date - Publication]) AND (humans[Filter]). For Scopus, the search strategy was (unicentric OR "unicentric castleman" OR UCD OR "single site" OR "localized castleman" OR "solitary castleman") AND ("castleman disease") AND (mesenteric OR mesentery OR "mesenteric lymph node" OR "mesenteric lymph nodes" OR "intra-abdominal" OR abdomen OR "abdominal cavity"). Filters were applied to include only human studies published between 1996 and 2026. Only English-language articles were considered. The protocol was registered on the research registry (UIN: reviewregistry2084). Two reviewers independently screened the studies; disagreements were resolved by consensus. Full-text articles from the retrieved references were assessed as per the inclusion and exclusion criteria.

Study Selection and Eligibility Criteria

Studies were eligible for inclusion if they reported cases of UCD occurring within the mesentery. Only reports describing disease confined to a single lymph node or a localized nodal region were considered. The diagnosis was required to be established through histopathological evaluation demonstrating characteristic features of CD, including hyaline vascular (HV), plasma cell (PC), or mixed variants. Furthermore, the lesion had to be explicitly described as arising from or involving the mesentery or mesenteric lymph nodes, with confirmation based on radiological imaging, operative findings, or pathological assessment. Only full-text articles published in the English language were included in the review. Studies were excluded if they involved MCD, reported CD occurring at anatomical sites other than the mesentery, were available only as conference abstracts without accessible full text, were published in languages other than English, or lacked sufficient clinical or pathological information necessary for meaningful data extraction and analysis. Studies meeting the inclusion criteria were reviewed, and data on age, sex, lesion size, location, histopathology, and management were extracted. We independently screened the references from PubMed and Scopus, initially by titles and abstracts. References that did not meet the inclusion criteria and for which full text was not available were excluded. Later, by reviewing full-text articles, articles that did not meet the inclusion criteria were excluded. The following variables: author and year of publication, patient demographic characteristics, tumor size, anatomical location within the mesentery, histopathological subtype (HV, PC, or mixed variant), treatment modality performed, duration of follow-up, and reported clinical outcomes were systematically extracted from each eligible study. The screening process was conducted in two stages. Initially, titles and abstracts identified through database searches were screened to exclude clearly irrelevant studies. Subsequently, the full texts of potentially eligible articles were reviewed to confirm eligibility based on the predefined inclusion and exclusion criteria. Discrepancies between the two reviewers were resolved through discussion and consensus. Because most available evidence consisted of individual case reports and small case series, a quantitative meta-analysis was not feasible. Therefore, the findings were synthesized using a systematic review approach.

Study Risk of Bias Assessment

The Joanna Briggs Institute (JBI) tool for case reports and case series was employed to evaluate the included articles [6]. Each case report was assessed across eight parameters, and each case series across ten parameters, with all items classified as “yes” (low risk of bias), “no” (high risk of bias), or “not applicable”. Two reviewers independently performed the risk-of-bias assessments, with disagreements resolved through discussion or consultation with a third reviewer. Risk-of-bias judgments were incorporated into the interpretation of the findings; studies with a higher risk of bias were interpreted with greater caution. As no meta-analysis was conducted, these assessments were used descriptively to contextualize the reliability and strength of the available evidence. Risk-of-bias data were recorded using standardized extraction sheets developed for this review.

Outcome Analysis

The primary outcomes of interest included: demographic distribution of patients with mesenteric UCD, including age and sex, tumor characteristics, specifically lesion size and anatomical location within the mesentery, histopathological subtype distribution (HV, PC, and mixed variants), treatment modalities employed, including complete surgical excision, bowel resection with anastomosis, laparoscopic excision, radiotherapy, or other surgical procedures, clinical outcomes, including postoperative recovery, recurrence rates, and overall prognosis during follow-up. All statistical analyses were performed using IBM SPSS Statistics for Windows, version 26.0 (IBM Corp., Armonk, NY, USA). Descriptive statistical methods were used to summarize the extracted data. Continuous variables such as age and tumor size were expressed as mean ± standard deviation, along with ranges when available. Categorical variables such as histological subtype, anatomical location, and treatment modality were reported as frequencies and percentages. Given the heterogeneity and case-based nature of the available data, the results were interpreted cautiously and presented primarily as descriptive trends rather than definitive comparative outcomes. We used descriptive statistics to report demographic details, clinical characteristics, histological type, and treatment modalities with means, frequencies, and percentages of variables.

Results

Literature Search

**Figure 1:**
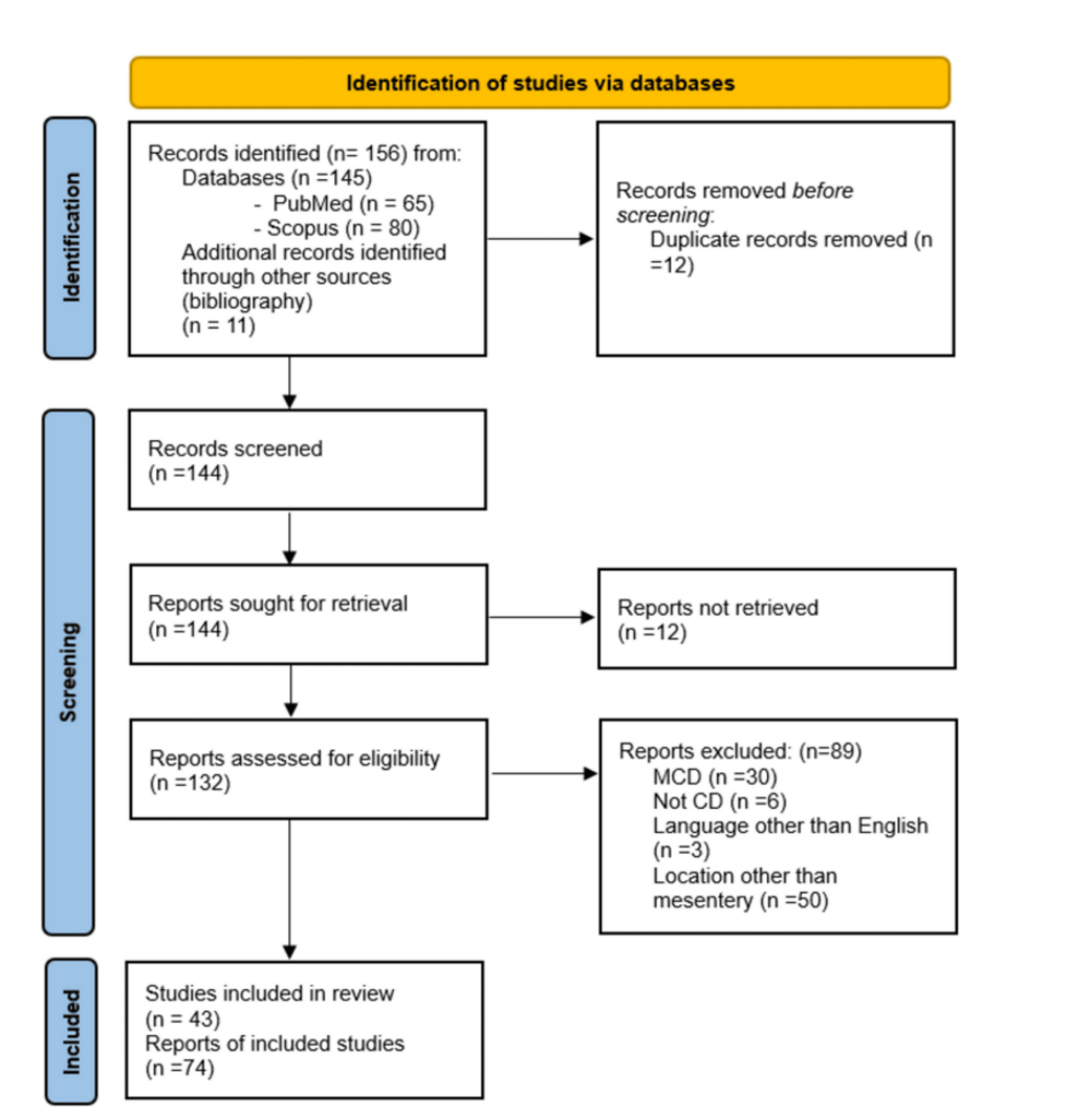
PRISMA flow diagram illustrating study identification, screening, eligibility assessment, and inclusion of studies in the systematic review. CD, Castleman disease; MCD, multicentric Castleman disease; PRISMA, Preferred Reporting Items for Systematic Reviews and Meta-Analyses.

A comprehensive literature search identified 145 records through PubMed and Scopus databases. An additional 11 articles were located from other sources (with bibliography), resulting in a total of 156 studies for initial screening. Of these, 12 records were excluded due to duplicate data. The remaining 144 articles were assessed for retrieval; 12 were excluded because they could not be retrieved. A total of 132 articles were assessed for eligibility, of which 89 articles were excluded: 6 were not related to CD, 30 were related to MCD, 50 did not involve the mesentery, and 3 were published in languages other than English. Ultimately, 43 studies met all criteria, out of which 74 reports were extracted and included in the final analysis (Figure 1).

Outcomes reported indicated favorable postoperative recoveries with minimal complications and low recurrence rates, reinforcing surgical excision as the mainstay of treatment. Given the reliance on descriptive case-level data, no comparative effectiveness evaluation was feasible. The data suggest a consistent clinical and pathological profile for UCD of the mesentery, emphasizing the role of surgery for definitive management. The synthesis highlights the heterogeneity in reporting, the inherent limitations of case-based evidence, and cautions regarding interpretation. It suggests areas for prospective data collection to better inform clinical guidelines for this rare entity.

Characteristics and Quality of Included Studies

A total of 74 cases of mesenteric UCD published between 1996 and 2026 were included in the final analysis following PRISMA-guided study selection (Table 1).

**Table 1:** Characteristics of included UCD cases presenting as mesenteric mass UCD, unicentric Castleman disease.

S. no.	Author (et al.)	Year	Age (years)	Sex	Size (cm)	Histology type	Location	Treatment	Follow-up duration	Outcome
1	Kimura et al. [7]	2002	16	M	4	Mixed	Mesenteric lymphoid hamartoma	Excision (autopsy)	No data	No data
2	Kimura et al. [7]	2002	57	F	17	Hyaline vascular	Small bowel mesentery	Excision (autopsy)	No data	No data
3	Kimura et al. [7]	2002	17	F	4.5	Plasma cell	Mesentery	Excision	No data	No data
4	Kimura et al. [7]	2002	20	M	6	Plasma cell	Mesenteric lymphoid mass	Excision	No data	No data
5	Kimura et al. [7]	2002	26	M	7	Plasma cell	Mesentery	Excision	No data	No data
6	Kimura et al. [7]	2002	8	M	6.5	Mixed	Mesenteric lymphoid hyperplasia	Excision	No data	No data
7	Kimura et al. [7]	2002	56	F	4.5	Plasma cell	Mesentery	Excision	No data	No data
8	Kimura et al. [7]	2002	2	F	4	Mixed	Ileocecal mesentery	Celiotomy	No data	No data
9	Kimura et al. [7]	2002	30	M	5	Plasma cell	Mesentery	Excision	No data	No data
10	Kimura et al. [7]	2002	11	F	6	Plasma cell	Mesentery	Excision	No data	No data
11	Kimura et al. [7]	2002	11	M	3	Hyaline vascular	Mesentery	Excision	No data	No data
12	Kimura et al. [7]	2002	19	F	6	Hyaline vascular	Mesentery	Excision	No data	No data
13	Kimura et al. [7]	2002	10	F	4	Plasma cell	Mesentery	Excision	No data	No data
14	Kimura et al. [7]	2002	11	M	8	Plasma cell	Mesentery	Excision	No data	No data
15	Kimura et al. [7]	2002	22	F	6	Hyaline vascular	Mesentery	Excision	No data	No data
16	Kimura et al. [7]	2002	30	M	4	Plasma cell	Mesentery	Excision	No data	No data
17	Kimura et al. [7]	2002	45	F	3	Plasma cell	Mesentery	Excision	No data	No data
18	Makiperna et al. [8]	2002	9	M	5	Mixed	Small bowel mesentery	Excision	12 months	No recurrence
19	Kimura et al. [7]	2002	60	F	4	Hyaline vascular	Mesentery	Excision	No data	No data
20	Kimura et al. [7]	2002	60	F	7	Hyaline vascular	Mesentery	Excision	No data	No data
21	Kimura et al. [7]	2002	29	F	5	Mixed	Mesentery	Excision	No data	No data
22	Kimura et al. [7]	2002	34	F	7	Hyaline vascular	Mesentery	Surgery	—	Good
23	Kimura et al. [7]	2002	44	M	6	Hyaline vascular	Mesenteric mass	Laparotomy	12 months	Cured
24	Wei et al. [9]	2004	12	M	9	Hyaline vascular	Mesentery	Surgical removal	—	Disease free
25	Kim et al. [10]	2005	13	F	4	Hyaline vascular	Mesentery	Resection and anastomosis	3 months	No recurrence
26	Papaziogas et al. [11]	2006	22	F	—	Hyaline vascular	Mesentery	Resection and anastomosis	12 months	No recurrence
27	Kaneko et al. [12]	2007	45	F	—	Hyaline vascular	Mesentery	Resection and anastomosis	24 months	No recurrence
28	Yang et al. [13]	2008	41	F	—	Plasma cell	Mesentery	Resection	18 months	No recurrence
29	Sari et al. [14]	2008	14	F	—	Mixed	Mesentery	Excision	12 months	No recurrence
30	El Demellawy et al. [15]	2009	33	F	1.8	Hyaline vascular	Small bowel mesentery	Excision	2 years	No recurrence
31	Bejjani et al. [16]	2009	29	F	4	Hyaline vascular	Jejunal mesentery	Excision	6 months	Anemia resolved, no recurrence
32	Toita et al. [17]	2009	5	M	—	Plasma cell	Mesentery	Excision	No data	No data
33	Al-Natour et al. [18]	2010	41	M	6×7	Hyaline vascular	Rectosigmoid mesentery	Lower anterior resection	12 months	No recurrence
34	De Vries et al. [19]	2010	67	F	—	Hyaline vascular	Mesentery	Radiotherapy (irresectable)	3 years	Stable disease
35	Reichard et al. [20]	2011	35	M	6	Plasma cell	Mesentery	Excision	18 months	No recurrence
36	Li et al. [21]	2011	12	F	3.8×3.7	Hyaline vascular	Mesentery	Resection	1 year	No recurrence
37	Ohta et al. [22]	2011	56	M	8	Hyaline vascular	Mesentery	Laparoscopic-assisted resection	12 months	Disease-free
38	Aslan et al. [23]	2011	43	F	6	Hyaline vascular	Ileal mesentery	Laparotomy	6 months	Good outcome
39	Shiote et al. [24]	2012	33	M	5	Hyaline vascular	Mesentery	Surgical removal	—	Well
40	Ma et al. [25]	2015	34	M	3	Hyaline vascular	Mesentery	Excision	1 year	No recurrence
41	Yang et al. [26]	2015	28	F	5.6	Hyaline vascular	Mesentery	Excision	2 years	No recurrence
42	Ang et al. [27]	2015	71	F	4×5	Hyaline vascular	Duodenojejunal mesentery	Pancreas-sparing duodenectomy with DJ anastomosis	1 year	No recurrence
43	Wang et al. [28]	2016	52	M	6	Hyaline vascular	Mesentery (with RCC)	Surgical resection	18 months	Healthy, no relapse
44	Bracale et al. [3]	2017	33	F	8×6×7	Hyaline vascular	Transverse mesocolon	Laparoscopic excision	1 year	No recurrence
45	Ozsoy et al. [29]	2018	55	F	7	Plasma cell	Small bowel mesentery	Resection and anastomosis	6 months	No recurrence
46	Kartal et al. [30]	2020	50	M	—	Hyaline vascular	Distal small intestine mesentery	Ileocecal resection and ileocolic anastomosis	1 year	No recurrence
47	Khanna et al. [31]	2024	42	F	6	hyaline vascular	Abdominal mesentery	Laparotomy excision	6 months	Asymptomatic
48	Khazaei Nasirabadi et al. [32]	2024	14	M	7	Hyaline vascular	Mesentery	Open resection	12 months	Complete recovery
49	Jahagirdar et al. [33]	2017	50	F	4.5	Hyaline vascular	Mesocolon	Laparoscopic excision	8 months	Disease free
50	Bhogal et al. [34]	2019	43	F	3	Plasma cell	Small bowel mesentery	Resection and anastomosis	No data	No data
51	Liedtke et al. [35]	2020	51	M	4×4×3	Hyaline vascular	Small bowel mesentery	Resection and anastomosis	12 months	No recurrence
52	Hamilton et al. [36]	2020	68	F	3	Hyaline vascular	Mesentery	Excision	1 year	No recurrence
53	Wu et al. [37]	2020	61	M	—	Hyaline vascular	Small bowel mesentery	Resection and anastomosis	2 years	No recurrence
54	Bernabei et al. [38]	2020	26	F	—	—	Mesentery	Excision	No data	No data
55	Lv et al. [39]	2020	55	M	6	Hyaline vascular	Mesentery	No data	No data	No data
56	Lv et al. [39]	2020	41	M	6	Hyaline vascular	Mesentery	No data	No data	No data
57	Lv et al. [39]	2020	57	M	5.4	Hyaline vascular	Mesentery	No data	No data	No data
58	Lv et al. [39]	2020	36	M	6.7	Hyaline vascular	Mesentery	No data	No data	No data
59	Lv et al. [39]	2020	33	F	8.2	Hyaline vascular	Mesentery	No data	No data	No data
60	Lv et al. [39]	2020	66	M	2.9	Hyaline vascular	Mesentery	No data	No data	No data
61	Lv et al. [39]	2020	63	F	5	Hyaline vascular	Mesentery	No data	No data	No data
62	Kadoura et al. [40]	2021	38	F	9	Hyaline vascular	Mesentery	Excision	6 months	No recurrence
63	Imamura et al. [41]	2022	40	M	5	Plasma cell	Mesentery	Resection and anastomosis	2 months	No recurrence
64	Ji et al. [42]	2024	30	M	6.5×5.2	Hyaline vascular	Mesentery	Resection and anastomosis	1 year	No recurrence
65	K A M et al. [43]	2024	36	F	3.2	Hyaline vascular	Small bowel mesentery	Resection and anastomosis	No data	No data
66	Chablou et al. [44]	2024	46	F	6×5	Mixed	Mesentery	Excision	12 months	No recurrence
67	Alessa et al. [45]	2025	10	F	4	Hyaline vascular	Mesentery	Complete resection	12 months	Disease free
68	Jain et al. [46]	2025	18	M	9	Mixed	Mesentery	En bloc excision	40 months	Disease free
69	Jain et al. [46]	2025	48	M	8	Hyaline vascular	Duodenojejunal flexure	En bloc excision	40 months	Disease free
70	Jain et al. [46]	2025	43	M	3	Hyaline vascular	Mesentery	Enucleation	16 months	Disease free
71	Jain et al. [46]	2025	34	M	3	Hyaline vascular	Mesentery	Robotic excision	7 months	Disease free
72	Jain et al. [46]	2025	58	M	4	Mixed	Mesentery	Excision	7 months	Disease free
73	Hua et al. [47]	2026	30	M	3.9	Hyaline vascular	Mesentery	Excision	12 months	No recurrence
74	Gupta et al. [48]	2026	21	M	4.3	Hyaline vascular	Right mesocolon	Excision	9 months	No recurrence

The majority of the included studies were single-case reports, with a few small case series describing more than one patient. All studies reported histopathologically confirmed UCD, which constituted the primary eligibility criterion for inclusion. The included reports originated from multiple geographic regions, reflecting the global distribution of published cases. Most studies provided detailed clinical descriptions, including patient demographics, imaging findings, operative management, and histopathological classification. However, there was variability in the completeness of reported data. While age and anatomical location were reported in most studies, variables such as tumor size, duration of follow-up, and postoperative outcomes were inconsistently documented. The methodological quality of the included studies was inherently limited due to the case-based nature of the available evidence. Most studies lacked standardized reporting of follow-up duration, outcome measures, and imaging characteristics. Additionally, the retrospective nature of case reports introduces potential publication bias, as unusual or successful clinical outcomes are more likely to be reported. Nevertheless, the majority of reports provided clear diagnostic confirmation through histopathology and adequate clinical description, supporting their inclusion in this systematic review. Overall, despite the limitations associated with case-report evidence, the included studies collectively provide valuable insights into the demographic patterns, clinical presentation, histopathological distribution, and treatment outcomes of mesenteric UCD.

Demographics and Clinical Characteristics

**Figure 2:**
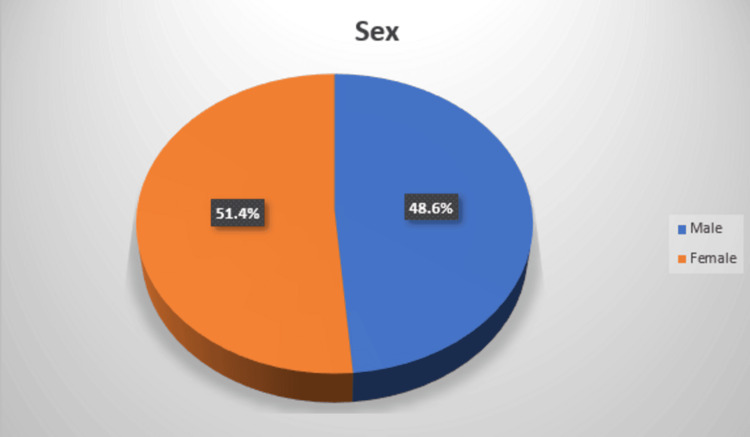
Sex distribution in reported mesenteric UCD cases This pie chart shows the sex distribution in reported mesenteric UCD cases, with 51.4% female and 48.6% male. UCD, unicentric Castleman disease.

Age data were available for 74 patients. The age ranged from 2 to 71 years, with a median of 34 years. Size data were reported for 65 patients, with 9 missing observations. The lesion size ranged from 1.8 cm to 17 cm, with a median size of 5 cm (Table 2). Patient sex distribution demonstrated near-equal representation between females 51.4% (n=38/74) and males 48.6% (n=36/74) among the 74 included cases (Figure 2).

**Table 2:** Age and tumor size characteristics of reported mesenteric UCD cases SD, standard deviation; UCD, unicentric Castleman disease. Tumor size was reported in 65 cases.

Variables	Number of cases (n)	Mean ± SD	Range
Age (years)	74	35.3 ± 17.9	2-71
Tumor size (cm)	65	5.51 ± 2.25	1.8-17

Location of Mesenteric Involvement

**Figure 3:**
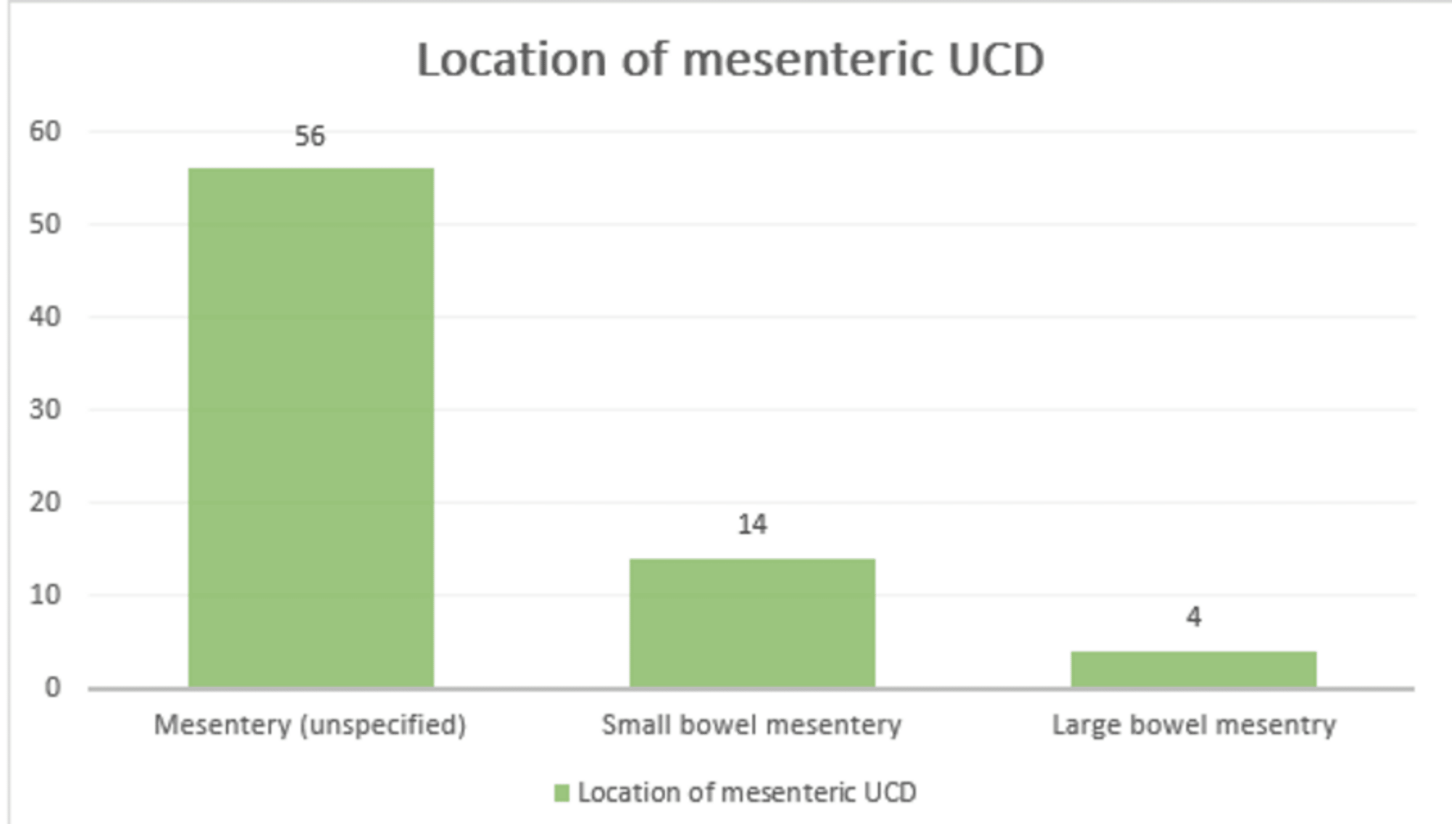
Anatomical distribution of mesenteric involvement in reported UCD cases The bar chart shows the location-wise distribution of mesenteric UCD. UCD, unicentric Castleman disease.

Among the 74 cases of mesenteric UCD, the specific location within the mesentery was detailed. The most common site was the general "mesentery" (unspecified), accounting for 75.68% (n=56/74) of cases. The small bowel mesentery was involved in 18.92% (n=14/74) of cases, followed by the large bowel mesentery at 5.41% (n=4/74) (Figure 3).

Histopathological Type

**Figure 4:**
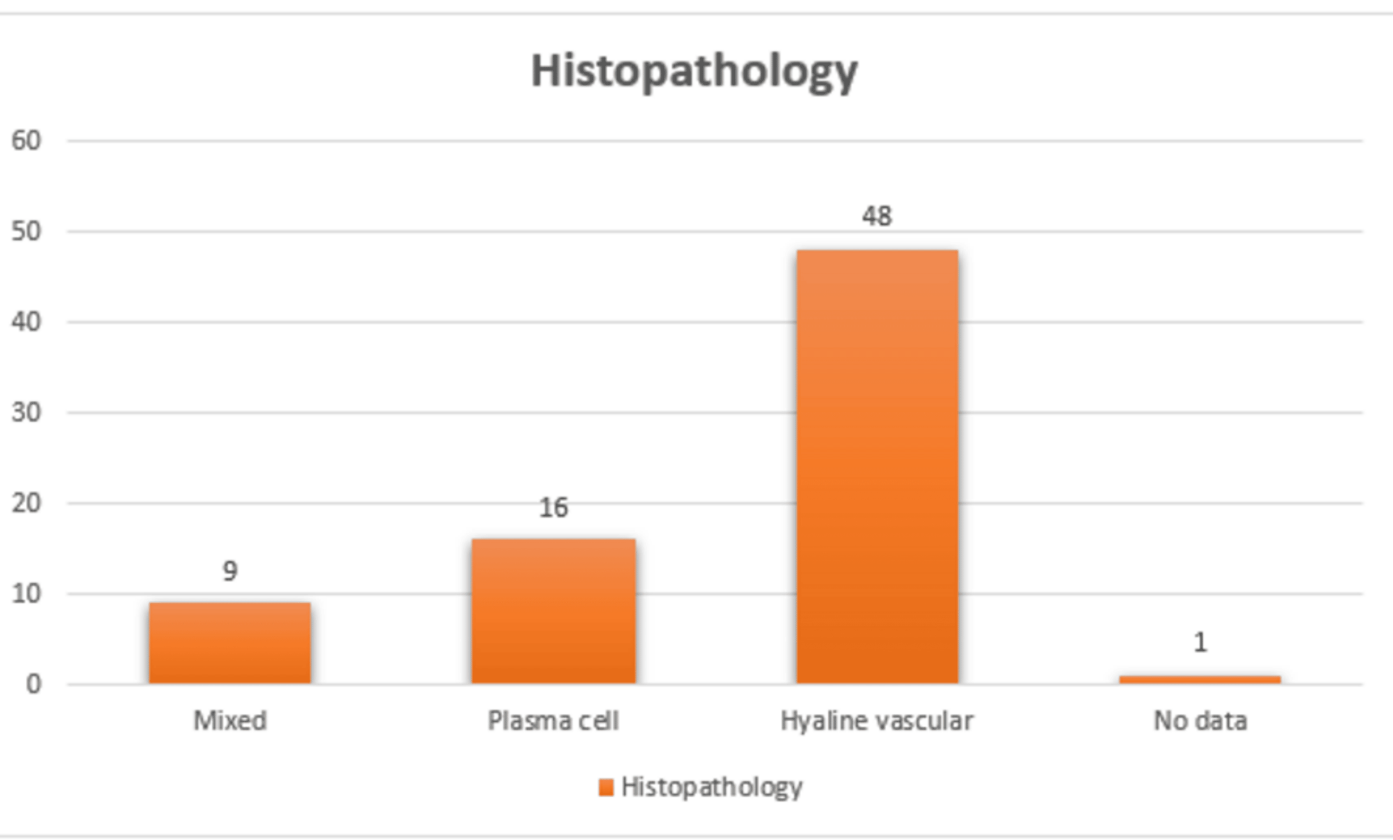
Histopathological subtype distribution in reported mesenteric UCD cases The bar chart depicts histological variants of mesenteric UCD. UCD, unicentric Castleman disease.

The histopathological evaluation revealed that the HV type was the most prevalent, representing 64.48% (n=48/74) of cases. The PC type was observed in 21.62% (n=16/74) of cases, while the mixed type accounted for 12.16% (n=9/74) of cases. For 1.35% (n=1/74) of cases, the histology type was not defined (Figure 4).

Treatment Modalities and Follow-Up

**Figure 5:**
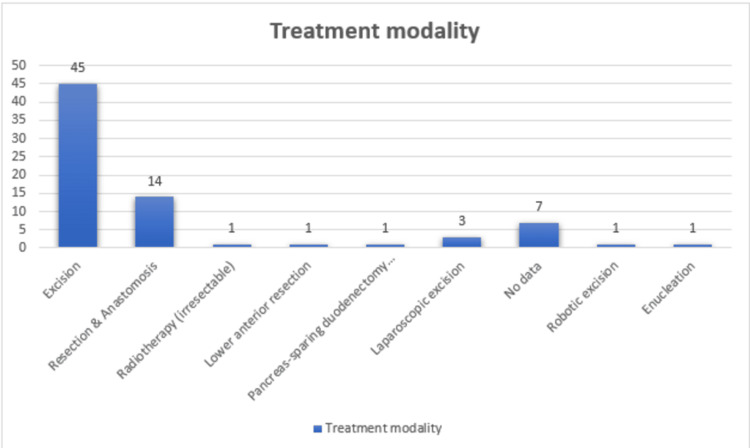
Treatment modalities used in reported mesenteric UCD cases The bar chart shows various treatment modalities, with excision being the most common (n=45), followed by resection and anastomosis (n=14). UCD, unicentric Castleman disease.

Various treatment modalities were employed for mesenteric UCD. Excision was the primary treatment approach, performed in 60.81% (n=45/74) of cases. Resection and anastomosis were applied in 18.92% (n=14/74) of cases. Laparoscopic excision accounted for 4.05% (n=3/74) of cases. Other less common treatments, each occurring in 1.35% (n=1/74) of cases, included low anterior resection, radiotherapy for irresectable disease, enucleation, robotic excision, and pancreas-sparing duodenectomy with duodenojejunal anastomosis (Figure 5).

Follow-up was available for only 58.1% (43/74) of cases, with a median duration of 12 months (range: 3-40 months), limiting recurrence assessment (0% in reported cases). This may overestimate favorable outcomes due to reporting bias. Clinical outcome data were available for 43 of 74 (58.1%) patients, of whom all remained free of recurrence (Table 3).

**Table 3:** Descriptive statistics of demographic, clinical, histopathological, and treatment modalities HV, hyaline vascular; PC, plasma cell.

Variables	Category	n (%)
Sex	Female	38 (51.4)
	Male	36 (48.6)
	Not reported	5 (6.8)
Location	General mesentery (unspecified)	56 (75.6)
	Small bowel mesentery	14 (18.9)
	Large bowel mesentery	4 (5.4)
Histopathology	HV variant	48 (64.8)
	PC variant	16 (21.6)
	Mixed variant	9 (12.1)
	Not defined	1 (1.3)
Treatment modality	Complete surgical excision	45 (60.8)
	Bowel resection with anastomosis	14 (18.92)
	Laparoscopic excision	3 (4.05)
	Low anterior resection	1 (1.35)
	Radiotherapy (unresectable tumor)	1 (1.35)
	Robotic excision	1 (1.35)
	Enucleation	1 (1.35)
	Pancreas-sparing duodenectomy with duodenojejunostomy	1 (1.35)
	No data	7 (9.46)
Clinical outcome	No data	31 (41.9)
	Follow-up >12 months	31 (41.9)
	Follow-up <12 months	12 (16.2)

Risk of Bias in Studies

**Figure 6:**
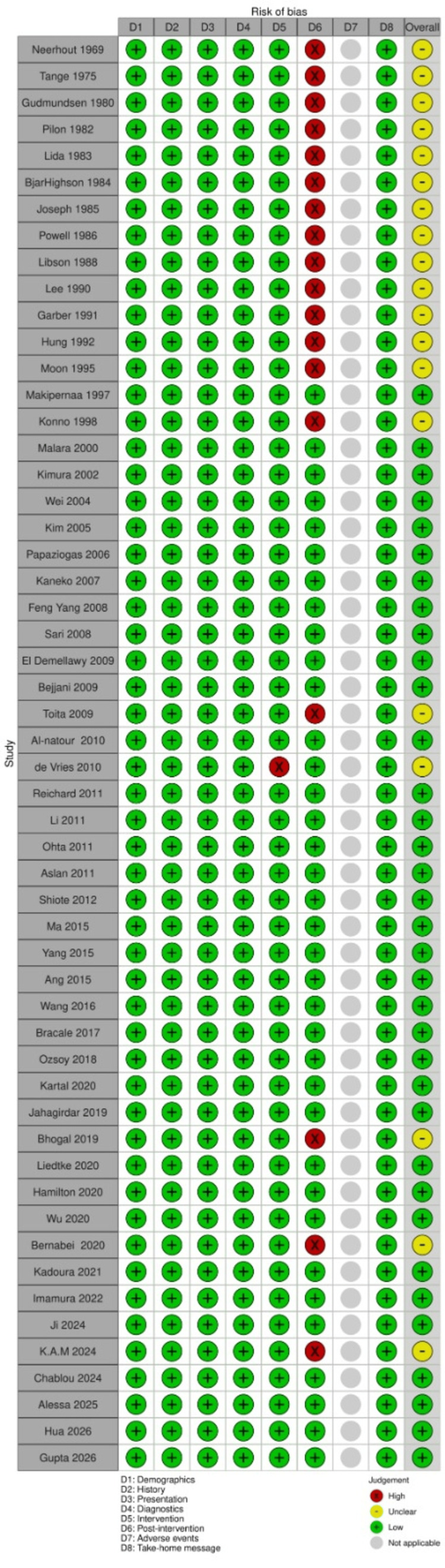
Risk of bias assessment according to the Joanna Briggs Institute (JBI) critical appraisal tool for case reports in systematic reviews

All case reports (n=55/100.00%) clearly described the patient’s demographic characteristics, history, current clinical condition at the time of presentation, diagnostic tests or assessment methods, and their results and intervention or treatment procedures. However, only 36 cases (65.45%) provided a clear description of the post-intervention clinical condition. Reporting of adverse events or unanticipated outcomes was considered not applicable for all studies. Finally, takeaway lessons were provided in all included reports (n=55/100.00%) (Figure 6).

**Figure 7:**
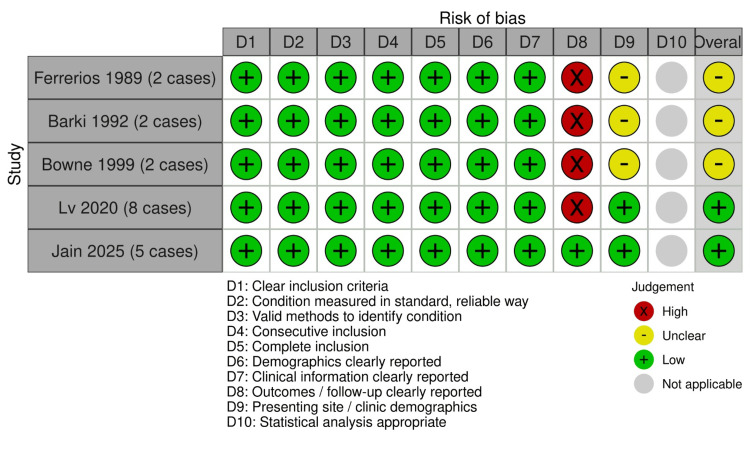
Risk of bias assessment according to the Joanna Briggs Institute (JBI) critical appraisal tool for case series in systematic reviews

Regarding case series, all cases (n=19/100.00%) clearly described the criteria for inclusion of participants and measured the condition in a standard, reliable way for all participants included in the case series. All included studies (n=19, 100%) employed valid diagnostic methods and demonstrated consecutive and complete inclusion of participants, with clear reporting of demographic and clinical characteristics. Similarly, most studies (n=14/42.1%) did not report the outcomes or follow-up results of cases, and most studies (n=6/31.5%) did not provide clear reporting of the presenting site/clinic demographic information (Figure 7).

Finally, statistical analysis was considered not applicable for all papers. Overall, the methodological quality of the included evidence was heterogeneous, with incomplete reporting of outcomes and follow-up data being the most frequent limitations across studies. These patterns of bias were taken into account when interpreting the findings of this review, particularly in studies with unclear or high risk of bias.

Discussion

CD represents a heterogeneous group of lymphoproliferative disorders with variable clinical behavior ranging from localized nodal enlargement in unicentric disease to systemic inflammatory illness in multicentric forms [1]. UCD of the mesentery represents an uncommon lymphoproliferative disorder often diagnosed incidentally or during evaluation of an unexplained abdominal mass. The findings from this systematic review, which incorporated 74 cases reported over nearly three decades, provide valuable insights into the demographic, clinical, histopathological, and therapeutic aspects of this rare entity.

Demography

The patients demonstrated a mean age of 35.3 years, indicating that mesenteric UCD predominantly affects young to middle-aged individuals, although cases ranged widely from early childhood to older adulthood. The mean lesion size was 5.51 cm, suggesting that these masses are typically of clinically appreciable size at presentation. The relatively large mean size may reflect delayed detection due to the deep anatomical location of the mesentery and non-specific symptomatology. Similar demographic trends have been noted in previous reports describing abdominal CD, where the disease often presents in otherwise healthy individuals undergoing evaluation for an unexplained abdominal mass [2].

One of the major challenges associated with mesenteric UCD is the difficulty in establishing a preoperative diagnosis. Radiological findings typically demonstrate a well-circumscribed hypervascular mass that can resemble other intra-abdominal neoplasms such as gastrointestinal stromal tumors, neuroendocrine tumors, lymphoma, or metastatic lymphadenopathy [3]. Because of this overlap in imaging characteristics, definitive diagnosis is usually achieved only after surgical excision and histopathological examination. Case reports describing mesenteric UCD have consistently emphasized this diagnostic challenge and the frequent initial suspicion of malignant pathology before histological confirmation [7-9].

Histopathology

Histopathologically, the HV variant remained the dominant subtype (64.8%), followed by PC (21.62%) and mixed variants (12.16%). The lower proportion of PC variant corresponds with the absence of systemic inflammatory manifestations typically observed in multicentric disease.

HV variant is characterized by small regressed germinal centers, concentric mantle zones, and increased vascular proliferation within lymphoid follicles [10]. The predominance of the HV subtype observed in our review (64.8%) is consistent with previous studies describing UCD, in which this subtype accounts for the majority of localized cases [11]. In contrast, the PC variant, which accounted for a smaller proportion of cases in our analysis, is more commonly associated with systemic manifestations and is more frequently observed in multicentric disease [12]. The mixed histological subtype represents an intermediate form and has been reported less frequently in the literature [13].

Location

Anatomically, the general mesentery (unspecified) accounted for the majority of cases (75.68%), followed by small bowel mesentery (18.92%) and large bowel mesentery (5.41%). Several authors have also described unusual presentations, including lesions involving the jejunal mesentery, mesocolon, and mesenteric root, occasionally requiring complex surgical procedures due to their proximity to major vascular structures [14-16].

Treatment Modalities and Follow-Up

Surgical excision remains the gold standard for managing UCD and is curative in most cases. Unlike MCD, which requires systemic therapy, UCD generally does not recur post-excision. Complete excision was performed in 60.81% of cases, while resection with anastomosis was required in 18.9%, usually when the lesion involved adjacent bowel. Minimally invasive approaches, including laparoscopic excision (4.05%), were reported in a smaller proportion of cases. Among patients with reported outcomes (n=43), zero recurrences were documented during available follow-up periods (median 12 months; range 3-40 months). This 0% recurrence rate at intermediate-term follow-up provides evidence supporting surgical excision as curative therapy for localized nodal disease. Numerous studies have demonstrated that complete resection results in excellent long-term outcomes with minimal risk of recurrence, supporting surgery as the definitive treatment modality for localized disease [17-19]. In some cases, bowel resection with primary anastomosis was required when the lesion was closely adherent to the intestinal wall or mesenteric vessel [20].

In rare situations where complete surgical resection is not feasible due to tumor location or involvement of critical vascular structures, radiotherapy has been described as an alternative therapeutic option. Reports of unresectable unicentric disease treated with radiotherapy have demonstrated favorable disease control, although such cases remain uncommon [21]. Nevertheless, the overall prognosis of UCD remains excellent when adequate local control is achieved.

Postoperative outcomes were overwhelmingly favorable, with very low recurrence rates reported across available follow-up data. Complete resection provides symptom relief and prevents potential complications such as mass effect and secondary compression of surrounding structures. Postoperative follow-up is necessary, although recurrence is rare in unicentric disease.

Limitations of this review include the inherent biases of case report data, such as heterogeneous reporting and potential publication bias toward successful surgical outcomes. Furthermore, the rarity of the condition and limited longitudinal follow-up data restrict the ability to make definitive prognostic or therapeutic recommendations beyond surgical excision.

Future studies, ideally prospective and multicentric, are warranted to better characterize the natural history of mesenteric UCD, explore adjunctive therapies in unresectable cases, and refine diagnostic algorithms incorporating advanced imaging and molecular markers.

## Conclusions

UCD of the mesentery is a rare pathology that predominantly presents as a localized mass, with a strong predilection for the HV subtype. Surgical resection stands as the primary and most effective treatment modality, often resulting in excellent patient outcomes. Awareness of this entity and its clinical-pathological features is critical for accurate diagnosis and avoiding misclassification. Continuing accumulation of clinical data will be essential to optimize management strategies and further elucidate the disease course.
